# Fatal Choking in Patients with Psychiatric Disorders: A Systematic Review of Forensic Evidence

**DOI:** 10.3390/diagnostics16182974

**Published:** 2026-09-14

**Authors:** Lucia Candela, Giorgia Rigano, Salvatore Ruben Spatola, Marija Čaplinskienė, Elvira Ventura Spagnolo, Gennaro Baldino

**Affiliations:** 1 Department of Biomedical and Dental Sciences and Morphofunctional Imaging, University of Messina, Via Consolare Valeria, 1, 98125 Messina, Italy; lucia.candela@studenti.unime.it (L.C.); giorgia.rigano@studenti.unime.it (G.R.); salvatorerubenspatola@gmail.com (S.R.S.); eventuraspagnolo@unime.it (E.V.S.); 2 State Forensic Science, Mykolas Romeris University, 08303 Vilnius, Lithuania

**Keywords:** choking, café coronary, psychiatric disorders, drugs, forensic pathology

## Abstract

**Background:** Fatal choking is a preventable cause of mechanical asphyxia that occurs more frequently among individuals with psychiatric disorders due to behavioural abnormalities, neurological comorbidities, and medication-related swallowing impairment. This review aims to highlight the clinical and forensic factors contributing to fatal choking and the importance of a multidisciplinary approach to its investigation. **Methods:** A systematic review was conducted according to PRISMA guidelines by searching PubMed, Scopus, and Web of Science. In total, 25 studies describing fatal choking in adults with psychiatric disorders and including medico-legal investigations were selected and analyzed. **Results:** Schizophrenia was the most frequently reported psychiatric condition, followed by bipolar disorder, depression, and dementia. Antipsychotics, antidepressants, anxiolytics, and other psychotropic drugs were recurrently associated with dysphagia and reduced airway protective reflexes. Food boluses represented the predominant obstructive material, although several unusual foreign bodies were documented. Virtopsy, autopsy, histological, and toxicological investigations consistently proved essential for confirming airway obstruction, reconstructing the mechanism of death, and identifying contributing clinical and pharmacological factors. **Conclusions:** Fatal choking in psychiatric patients is a multifactorial event requiring early risk assessment, preventive strategies, and comprehensive forensic evaluation to improve patient safety and ensure accurate determination of the cause of death.

## 1. Introduction

Choking is a significant and widespread health and safety problem across the lifespan [1]. From a pathophysiological perspective, choking is a form of mechanical asphyxia caused by partial or complete obstruction of the airway by solid or semi-solid material. Although food-bolus asphyxia is often framed within the concept of “café coronary,” fatal choking should be understood more broadly as a heterogeneous event that may reflect clinical vulnerability, environmental context, and circumstantial factors [2,3,4]. Clinical signs of choking include gagging, inability to speak, cry, or breathe, anxiety or agitation, loss of consciousness, and cyanosis [1]. It occurs when the airway becomes obstructed, most commonly between the pharynx and the tracheal bifurcation. Airway obstruction may be partial or complete. Partial obstruction allows limited airflow and is typically associated with wheezing and forceful coughing as the individual attempts to expel the obstructing material. By contrast, complete obstruction prevents effective airflow and rapidly leads to hypoxia, loss of consciousness, and death if not promptly relieved [2]. Obstruction may result from several causes, including foreign bodies such as metal or plastic objects, food material (for example, vomitus or a food bolus), and acute obstructive conditions. The latter include glottic or laryngeal edema caused by hypersensitivity reactions, exposure to irritant fumes, inhalation of hot gases, or infection. In addition, chronic benign lesions, such as laryngeal cysts, polyps, lingual tonsil hypertrophy, and hemangiomas, may narrow the upper airway and increase the risk of choking [3]. Some populations are particularly vulnerable to choking, including children, older adults, and individuals with psychiatric disorders, neurological disease, or intellectual disability. In these groups, risk is related not only to the underlying disorder but also to pharmacological treatment and abnormal eating or swallowing behaviors, such as rapid food intake and poor assessment of bolus size during mastication [4]. Among the accidental causes food gullet choking is one of the most common causes of laryngeal obstruction, a phenomenon commonly referred to as “café coronary.” The term, introduced by Haugen in 1963, describes sudden and unexpected death during a meal as a result of accidental airway occlusion by food [3]. Homicidal deaths due to choking are relatively rare and are often associated with gagging. Cases of homicide simulating accidental choking are reported in the literature [5]. Whereas suicidal choking, although uncommon, has been documented. In this context, an atypical but relevant behavior is deliberate foreign body ingestion (DFBI), defined as the intentional, non-accidental ingestion of non-nutritive objects, usually in the setting of parasuicidal behavior [6]. The mechanisms underlying elevated asphyxiation risk in psychiatric patients and ID are multifactorial, involving cognitive, behavioural, physiological, and iatrogenic components. A vulnerability to choking has also been reported in the presence of primary psychiatric disorders, particularly schizophrenia as well as progressive or structural neurological conditions. These conditions may impair swallowing coordination, weaken the airway’s protective reflexes, and reduce overall neuromuscular control, thereby increasing susceptibility to choking. This review aims to provide clear educational and practical value to forensic pathologists, clinicians, psychiatrists, and other healthcare professionals involved in the management and investigation of fatal choking cases, emphasising the importance of a multidisciplinary approach in establishing airway obstruction, reconstructing the mechanism of death, and identifying contributing clinical and pharmacological factors.

## 2. Material and Methods

**Figure 1:**
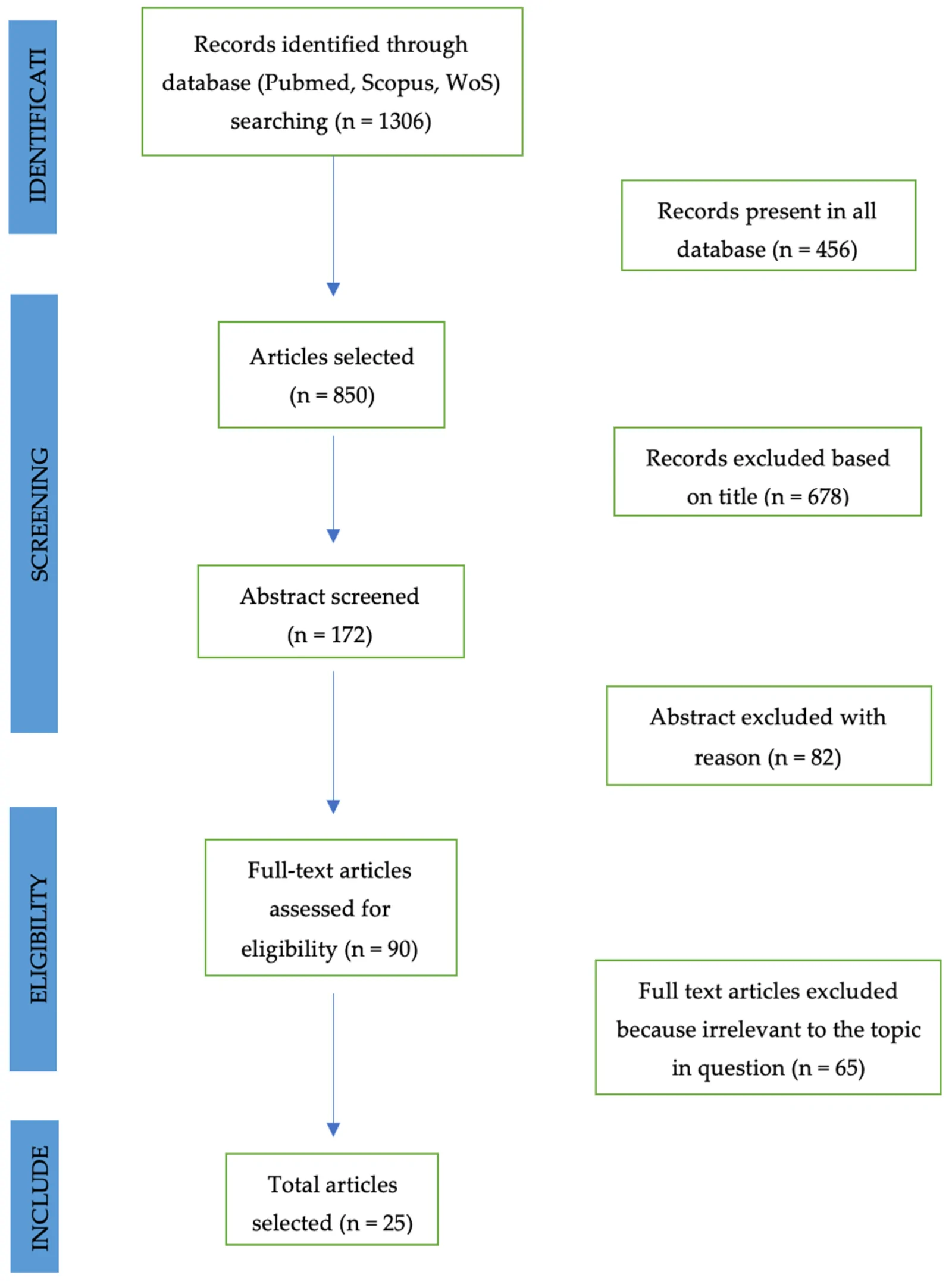
Flowchart of the article selection process.

This review was conducted using searches of PubMed (https://pubmed.ncbi.nlm.nih.gov/, accessed on 11 November 2025), Scopus, and Web of Science ((https://www.webofscience.com/wos/author/, accessed on 11 November 2025) databases. The review was carried out in accordance with PRISMA guidelines (File S1) [7]. The inquiry strategy was performed by combining the terms: “choking” to the keywords “Psychiatric”, “Mental disorder”, “death”, “mortality”, “fatal”; “food”, “Mental disorder”, “foreign body”, “swallowing”, “suffocation”, “smothering”, “adult” in various combinations of search strings through the Boolean operators “AND” and “OR”. Preliminary research led to a total of 1279 articles found on 246 records in PubMed, 978 in Scopus, and 55 in Web of Science. Studies were then assessed according to the following inclusion criteria: (a) full-text articles published in English; (b) primary research studies; (c) reports concerning fatal choking events; (d) cases in which at least one medico-legal investigation was performed, with attention to scene inspection, autopsy, histology, toxicology, or radiology. The following exclusion criteria were considered in selecting the articles to be enrolled: exclusion criteria: (a) reviews, letters, commentaries, editorials, and meeting or conference abstracts; (b) studies reporting only non-fatal events; (c) studies in which psychiatric disease could not be inferred, or in which results for individuals with psychiatric disorders were not reported separately; (d) studies exclusively involving pediatric populations; (e) studies addressing the role of asphyxia in sexual practices. Titles and abstracts were screened to exclude records clearly outside the scope of the review. Potentially relevant articles were then retrieved in full and assessed for eligibility on the basis of the predefined criteria. This led to the selection of a total of 25 articles (Figure 1) including case reports, case series (referred to as articles discussing ≥3 cases). All selected articles are summarized in Table 1 according to authors, title, year and type of publication [2,3,8,9,10,11,12,13,14,15,16,17,18,19,20,21,22,23,24,25,26,27,28,29,30].

According to what was decided during the planning stage of the article, a data extraction form was created, piloted, and refined by the authors. Data extracted included sample size and demographic characteristics, psychiatric diagnosis or antipsychotic possibly causing the swallowing impairment, associated medical conditions, medico-legal investigations that were performed, autopsy findings, circumstantial data, histological, toxicological, radiological investigations.

The extracted information was then tabulated in an Excel database. Results were collated, summarised and presented as a tabulation of key variables and a narrative synthesis of the included studies.

## 3. Results

All cases were categorized in Table 2 and Table 3 according to the following indicators: number of cases analyzed, personnel information (age, gender, psychiatric disease and other disease, drugs/medicaments) heading the survey at the recovery site, circumstantial data, investigations carried out (autopsy, histopathology, radiology, toxicology, others), witnessed death.

### 3.1. Psychiatric or Neurological Diseases

In the included literature, schizophrenia was the most frequently reported psychiatric diagnosis among individuals who died from choking [2,3,8,9,10,11,16,17,18,19,21,28]. It is followed, with progressively lower prevalence, by bipolar disorder [14,21,22,28], major depressive disorder [21,29], anxiety disorder [3,26], schizoid personality disorder [24], schizo-affective disorder [13,20], personality disorder subtypes (including paranoid [13], histrionic [29], and borderline [15] presentations) and, less frequently, obsessive–compulsive disorder [18]. Because the evidence base consisted mainly of case reports and case series, these findings should be interpreted descriptively rather than as estimates of disease-specific incidence or risk.

Beyond primary psychiatric disorders, choking vulnerability was also reported in the presence of progressive or structural neurological disease. In particular, dementing conditions, such as Alzheimer’s disease [3,18,27], senile dementia [3,21,23,31,32], and Parkinson’s disease, appear to substantially increase vulnerability. An elevated likelihood of choking was also observed in individuals affected by epilepsy [33], encephalopathy [33], acquired brain injury, bulbar palsy [30], ataxia [33], perinatal asphyxia [20], ischemic stroke [20], and intellectual disability [3,18,20,21,25,27]. These disorders may impair swallowing coordination, weaken protective airway reflexes, and reduce overall neuromuscular control, thereby increasing susceptibility to choking.

### 3.2. Drugs

Medication-related choking emerged as a clinically relevant issue across the reviewed cases. The drug classes most frequently reported were antipsychotics, anxiolytics, antidepressants, antiepileptic drugs, anticholinergic agents, and lithium [14,15,16,20,22,27,30,31]. These medications may contribute to risk through sedation, impaired coordination, xerostomia, dysphagia, and attenuation of protective airway reflexes, particularly in older adults and in patients with neurological comorbidity or polypharmacy. Consequently, the interplay between disease-related factors and medication-induced side effects should be carefully considered when evaluating choking risk in this patient population.

### 3.3. Survey at the Site of Recovery and Circumstantial Data

Inspection reports and circumstantial information were not reported uniformly across studies, which limited comparison between cases.

Among the cases for which this information was available, 17 deaths were witnessed and 10 were classified on the basis of inspection findings only. A “witnessed death” was defined as a case in which at least one person directly observed the entire sequence of events while the individual was still alive. In contrast, an “inspection” case refers to situations in which the deceased was discovered after death had already occurred, without any direct observation of the terminal phase. Witnessed cases often described sudden collapse during a meal, ineffective coughing, cyanosis, agitation, and rapid clinical deterioration [12,20].

A substantial proportion of individuals were hospitalized or institutionalized at the time of death, often in psychiatric facilities or nursing homes. Several reports also described eating-related behaviors that may have increased risk, including rapid food intake, poor mastication, cramming of the mouth, or reduced supervision during meals.

### 3.4. Foreign Body

Autopsy reports documented a wide variety of foreign bodies causing airway obstruction. These findings ranged from common food items to highly unusual and unexpected objects. The most frequently encountered materials, and arguably the least surprising, included ordinary dietary components such as meat, fruit, vegetables, eggs, apricots, pasta, peanut butter, bread, potato chips, biscuits, pizza, and cake [2,16,17,18,20,21,33]. More atypical findings were also documented. These comprised two cases involving a whole fish [11,22], as well as non-food items such as a bottle cap [16], a handkerchief [10], components of a nebulizer device [13], paper [15], nappies [32], metallic pins [29], and pharmaco-bezoars (a bezoar is an aggregate or conglomerate of undigested foreign material) [26]. Additional extraordinary cases included the presence of faecal material [23], fragments of stone or wood [25], and, notably, one case involving an entire raw cat [14]. This heterogeneity highlights the need for careful forensic reconstruction of each event.

### 3.5. Histological and Autoptic Findings

Histological findings primarily involved the upper aerodigestive tract and the lungs. Reported upper-airway changes included mucosal erosion, hemorrhage, congestion, edema, and acute inflammatory infiltrates, findings that are consistent with local trauma caused by impacted material [34]. Pulmonary histopathology revealed findings consistent with acute respiratory distress and shock [24]. The lungs showed passive congestion, with scattered areas of recent interstitial hemorrhage. Alveolar spaces were frequently overdistended, accompanied by rupture of interalveolar septa. Widespread alveolar edema and features of pulmonary vasculopathy—including pulmonary edema and interstitial emphysema—were documented [19,30]. In several cases, amorphous granular material was identified within alveolar lumina, along with endo-alveolar hemorrhages [9]. Small foreign particles were detected in the bronchi of small and very small caliber, as well as within alveolar cavities [20]. Small subpleural and subepicardial petechiae were occasionally noted [2].

Additional findings included increased bacterial overgrowth attributable to postmortem autolysis and aspiration phenomena. Foci of bronchopneumonia, composed predominantly of monocytes and polymorphonuclear leukocytes, were observed, together with an increased number of multinucleated foreign body giant cells, possibly indicative of prior aspiration events. Leukocytic infiltration of the bronchi, intra-alveolar and interstitial hemorrhages (suggestive of blood aspiration and parenchymal bleeding), and diffuse arteriolar microthromboembolism were also described [27,28]. In the brain, some reports described vascular congestion and edema [28].

Gastric findings were non-specific and included superficial gastritis and, in a few cases, fibrinopurulent deposits along the external gastric wall [24,25,26,27,28,29]. Finally, a pattern of multiorgan congestion was frequently observed [10], consistent with systemic hypoxia and terminal circulatory failure.

The findings of the present investigation indicate that foreign materials were predominantly identified within the upper aerodigestive tract, including the oral cavity, nasopharynx, oropharynx, laryngeal inlet, and the oesophageal lumen [9,11,12,13,14,15,20]. In several instances, the foreign body maintained the epiglottis in an open configuration [35]. Specifically, 10 cases involved a supraglottic location, 8 were confined to the infraglottic region, and 15 demonstrated a combined supra and infraglottic distribution [17]. Examination of the laryngeal mucosa revealed impacted alimentary debris admixed with frothy fluid. In the upper oesophageal lumen, a necrotizing ulcerative lesion was observed, extending into the longitudinal layer of the muscularis interna and surrounded by an acute exudative inflammatory infiltrate [25].

Skeletal findings included previous fractures involving the right superior laryngeal horn [28]; more generally, the superior cornua of the thyroid cartilage were found to be disrupted. Fresh haemorrhagic infiltration was also documented at the origins of the sternocleidomastoid muscles [29]. In some cases, obstructing material extended distally to the tracheal bifurcation, main bronchi, lobar bronchi, and smaller airways, confirming aspiration of fragmented material [2,3,4,5,6,7,8,9,10,11,12,13,14,15,16,17,18,19,20,21,22,23,24,25]. Histopathological assessment demonstrated focal mild-to-moderate acute and subacute tracheitis with inflammatory involvement reaching the perichondrium. Abundant foam was present within both the trachea and pulmonary parenchyma [27].

Additional thoracic findings included apical pleural adhesions, fluid blood within the pulmonary vasculature in the absence of thrombi or clots [27], and multilobular confluent infiltrates characterized by multiple greyish foci and areas of consolidation bilaterally. On gross examination, the lungs appeared increased in volume and exhibited an emphysematous consistency upon palpation [16].

### 3.6. Toxicological Analyses

Toxicological analyses were systematically performed on available biological matrices, including peripheral blood, gastric contents, urine, and vitreous humour [34]. These investigations were aimed not only at identifying the presence of drugs, toxic substances, or other chemical agents, but also at quantifying their concentrations in order to determine whether levels fell within therapeutic ranges or reached potentially toxic or lethal thresholds. Particular attention needs to be paid to the gastrointestinal tract, as drug concentrations detected in gastric contents may provide useful information regarding the interval between ingestion and death. For instance, the detection of minimal residual amounts of clozapine in the stomach can contribute to reconstructing the timing of administration relative to the fatal event [10]. Toxicological findings also play an essential role in corroborating preliminary hypotheses formulated during the scene investigation and external examination of the body, especially in cases of suspected self-harm. In our study, the substances most frequently implicated included overdoses of clozapine [10,11,28], elevated concentrations of venlafaxine and quetiapine, as well as the presence of oxycodone, metoclopramide, topiramate, lorazepam, loratadine, escitalopram [27] and levodopa [20]. In some cases, toxicological testing revealed lethal concentrations of quetiapine and paracetamol, alongside supratherapeutic levels of lamotrigine and metformin [26]. Additionally, markedly increased blood alcohol levels [3,12,18,22,23,24,25,26,27,28,29,30] and the presence of tricyclic antidepressants [24] were commonly observed. Overall, these results highlight the importance of comprehensive toxicological assessment in clarifying both the mechanism of death and the potential contribution of pharmacological agents in complex medico-legal cases.

## 4. Discussion

Although food-bolus asphyxia is often framed within the concept of “café coronary,” fatal choking should be understood more broadly as a heterogeneous event that may reflect clinical vulnerability, environmental context, and circumstantial factors [12,13,14,15,16,17,36].

Despite its clinical relevance, food-bolus asphyxia remains underrepresented in the epidemiological and preventive literature, especially outside pediatric and geriatric settings [37].

Previous studies have reported a markedly increased choking-related mortality in psychiatric populations [38], particularly in institutional settings, with estimates ranging from approximately 8-fold to 43-fold higher than in the general population [39,40]. The data collected in our study suggest that schizophrenia represents the psychiatric diagnosis most strongly associated with choking events. However, the literature also points to an important contribution from bipolar disorder, depressive disorders, anxiety disorders, personality disorders, and schizoaffective disorder, as well as from neurological disease and intellectual disability. Taken together, these findings support a multifactorial model in which psychiatric symptoms, cognitive impairment, dysphagia, motor dysfunction, and environmental factors interact to increase risk. Notably, schizophrenia, but also depression or bipolar affective disorder have been identified as risk factors for aspiration pneumonia among stroke survivors [41]. The risk of choking is also significantly influenced by the presence of progressive or structural neurological disorders; neurodegenerative conditions, such as Alzheimer’s disease, senile dementia, and Parkinson’s disease, were particularly associated with increased vulnerability. A higher incidence of choking was also documented among individuals with epilepsy, encephalopathy, acquired brain injury, bulbar palsy, ataxia, perinatal asphyxia, ischemic stroke, and intellectual disability. Taken together, these neurological impairments may disrupt the coordination of swallowing, weaken protective airway reflexes, or reduce overall neuromuscular control, thereby increasing susceptibility to choking events. As well as psychiatric diseases, intellectual disability (ID) is a well-established risk factor for dysphagia, with prevalence and severity increasing in parallel with the degree of cognitive impairment [41]. Choking represents a major cause of death among adults with ID, accounting for a substantial proportion of premature mortality. Individuals with ID frequently present with oral and pharyngeal phase impairments, including abnormal oral tone, immature or inefficient chewing, limited tongue control, reduced oral sensation, and compromised bolus formation. Neurological involvement affecting oral musculature is common and contributes to dysphagia. Oral stage dysfunction combined with cognitive impairment significantly increases asphyxiation risk [1].

Cognitive impairment directly affects mealtime safety through poor bolus judgment, impaired pacing, and reduced awareness during eating. Adults with schizophrenia, depressive states, bipolar disorder and ID are more likely to demonstrate impulsivity, rapid eating (tachyphagia), and cramming or overstuffing the mouth. These maladaptive eating strategies have been identified as strong and consistent predictors of choking and near-fatal asphyxiation events. Poor bolus judgment, impulsivity, rapid eating, food cramming, premature spillage of the bolus into the pharynx, poor dentition, seizure disorders, Parkinsonian features, and inadequate supervision may all compromise mealtime safety. Institutional factors, including hurried feeding practices or insufficient monitoring, may further amplify risk [4,40,42]. For this reason, systematic assessment of choking and aspiration risk is recommended in psychiatric settings, particularly when making clinical decisions regarding patient privileges or discharge [43].

Psychotropic treatment may further increase choking risk through several mechanisms. Indeed, polypharmacy and psychotropic medication use may further increase swallowing risk through several mechanisms: sedation and reduced alertness, motor impairment, xerostomia (dry mouth), and medication-related dental deterioration. An association has been observed between the number of medications and the frequency of swallowing difficulty indicators [42]. Medication-related choking constitutes a clinically significant issue and appears to be linked to specific classes of pharmacological agents. Our review demonstrates that the medications most commonly associated with choking episodes included anxiolytics, antidepressants, antipsychotics, antiepileptic drugs, anticholinergic agents, and lithium. These treatments are routinely prescribed for psychiatric and neurological conditions, populations that already exhibit an intrinsically elevated baseline risk. Notably, the occurrence of choking in these patients cannot be explained solely by the natural course of the underlying disorder. Drug-related adverse effects may play a contributory role, particularly when they interfere with swallowing physiology. Complications such as dysphagia, excessive sedation, impaired motor coordination, and attenuation of protective airway reflexes may significantly increase vulnerability consistent with Fioravanti et al. [44]. Drug-induced dysphagia mechanisms are multifactorial. Dopamine D2 receptor antagonism in the nigrostriatal pathway, typical of many antipsychotics such as clozapine, chlorpromazine, and periciazine, may induce extrapyramidal symptoms (EPS), parkinsonism, dyskinesia, and involuntary oromotor activity [20,45,46,47]. Pathophysiologically, antipsychotic-induced dysphagia primarily affects the oral and pharyngeal phases through impaired lingual coordination, delayed swallow reflex, incomplete laryngeal elevation, reduced glottic protection, and pharyngeal pooling mechanisms closely linked to aspiration and asphyxia. Acute dystonia (more common with high-potency first-generation agents such as haloperidol) and tardive dyskinesia (bucco-lingual-masticatory syndrome) represent additional extrapyramidal contributors. Five dysphagia-related mechanisms in tardive dyskinesia have been described: impaired protective reflexes, choreiform oromotor incoordination, pharyngeal/upper esophageal dyskinesia, delayed laryngeal closure, and respiratory–swallow discoordination. Finally, pronounced anticholinergic burden may induce severe xerostomia and inhibit esophageal peristalsis, whereas excessive H1-mediated sedation, particularly with clozapine, may blunt laryngeal reflexes and facilitate aspiration pneumonia in frail elderly populations. Moreover, first- generation antipsychotic can result in sedation, sialorrhea, constipation, and seizures, which can increase the risk of aspiration pneumonia [48]. Collectively, these findings underscore the complex pharmacodynamic interplay underlying dysphagia and choking risk in psychiatric and neurologically vulnerable individuals [26]. Antihistaminergic (H1) and anticholinergic (muscarinic) effects contribute to sedation, xerostomia, reduced esophageal motility, delayed gastric emptying, and impaired bolus transport, particularly in older adults. Benzodiazepines may suppress brainstem swallowing regulation, leading to pharyngeal retention, cricopharyngeal incoordination, aspiration, and pneumonia. Antiparkinsonian agents (e.g., levodopa/carbidopa, amantadine, bromocriptine, rasagiline) and antiepileptics such as oxcarbazepine have also been associated with oropharyngeal dysphagia (OD), although disentangling drug effects from underlying neurological disease remains challenging [46]. Appetite stimulation and tachyphagia induced by some antipsychotics may further increase choking risk, especially in elderly or chronically institutionalized patients with poor dentition and impaired gastrointestinal motility [40,49]. Combined antidopaminergic and anticholinergic exposure, advanced age (>50 years), and higher dosages appear to synergistically heighten risk [44].

Although choking is predominantly accidental, self-inflicted events have also been reported. In these cases, the behavior is often framed as deliberate foreign body ingestion and may occur in the setting of psychosis, severe personality disorder, intellectual disability, pica, or institutionalization. Motives may range from parasuicidal intent to impulsive self-injury or the pursuit of secondary gain. These cases are uncommon but clinically and forensically important because intent may be difficult to reconstruct. In the context of psychotic illnesses, for example, such conduct may emerge as a direct consequence of hallucinations or firmly held delusional convictions that influence the individual’s actions. Similar behaviours have also been described in people with intellectual disabilities, where they may present in the form of Pica disorder. Additionally, they are reported within institutional settings, including correctional facilities and long-term psychiatric units, where the behaviour can, in some cases, serve instrumental purposes and be enacted to obtain secondary gain. A strong association has further been noted with severe personality disorders. In these cases, the acts may occur impulsively or follow a premeditated and repetitive pattern, often proving particularly challenging to manage and demonstrating limited responsiveness to conventional therapeutic interventions [6]. Beyond accidental ingestion and suicidal behavior, less common motives include substance abuse and eating disorders. In this context, Pica disorder represents a qualitative eating disorder characterized by the persistent ingestion of non-nutritive substances. The term derives from the Latin designation of the magpie (Pica pica), reflecting the bird’s indiscriminate feeding behavior. Clinical consequences vary according to the type, size, and number of ingested objects. Wooden materials, ranging from toothpicks to larger branches, may cause gastrointestinal or airway obstruction and perforation. This specific manifestation, termed xylophagia, constitutes a rare but potentially life-threatening condition [28].

The reviewed autopsy reports documented a striking variety of obstructing materials, ranging from ordinary food items to highly unusual non-food objects. This heterogeneity reinforces the need for meticulous scene investigation, careful airway dissection, and correlation between circumstantial, clinical, and postmortem findings [50]. Less typical cases were also recorded. These included two instances involving a whole fish, as well as various non-edible objects such as a bottle cap, a handkerchief, components of a nebulizer device, paper, nappies, metallic pins, and pharmacobezoars [26,32]. Additional extraordinary findings comprised faecal material, fragments of stone or wood, and, notably, one case in which an entire raw cat was recovered.

Klein et al. [28] described a case in which postmortem computed tomography identified angular foreign bodies within the oral cavity and pharyngeal region, together with extensive soft-tissue emphysema. Autopsy subsequently confirmed death due to airway obstruction and aspiration of blood and foreign material caused by the insertion of wooden and reed fragments. The case illustrates the added value of imaging in documenting anatomical relationships before dissection and in supporting reconstruction when the mechanism of injury is not immediately clear.

Morio Iino et al. [30] proposed a three-group classification based on postmortem CT findings. Type 1 is characterized by a foreign body located between the oral cavity and the oropharynx, with the epiglottis in its normal position. Type 2 involves a foreign body immediately above the epiglottis, displacing it posteriorly and obstructing the airway. Type 3 refers to a foreign body at the level of the laryngeal inlet, pushing the epiglottis anteriorly. This framework supports the role of postmortem CT as a useful adjunct for documenting the location of the bolus before autopsy-related displacement can occur.

An integrated approach combining scene investigation, postmortem imaging, autopsy, histology, toxicology, and exclusion of third-party involvement allows a more robust medico-legal interpretation [49,50,51]. At present, however, autopsy remains the reference standard for definitive identification and localization of foreign bodies and for assessment of associated injuries.

This review has several limitations. The available evidence consists mainly of case reports and small case series, with heterogeneous reporting of psychiatric diagnoses, medications, circumstantial data, and postmortem findings. Accordingly, the results should be interpreted descriptively and should not be used to infer incidence, causality, or effect size.

## 5. Conclusions

Fatal choking is a complex and multifactorial event at the intersection of clinical medicine, psychiatry, neurology, pharmacology, and forensic science. Although often perceived as an accidental and isolated event, the data demonstrate that it frequently reflects an intricate interplay between individual vulnerability, environmental context, behavioural patterns, and iatrogenic influences. Psychiatric disorders, particularly schizophrenia, along with intellectual disability and progressive neurological diseases, substantially amplify the risk, especially when compounded by maladaptive eating behaviours, institutional dynamics, and polypharmacy. Pharmacological treatment, while indispensable for the management of severe mental illness, may paradoxically contribute to swallowing dysfunction through mechanisms involving sedation, extrapyramidal symptoms, impaired coordination, xerostomia, and reduced airway protective reflexes. The cumulative burden of these effects, particularly in older or medically fragile individuals, underscores the need for systematic risk assessment and careful therapeutic balancing. From a forensic perspective, the remarkable heterogeneity of foreign bodies, the variability of histopathological findings, and the occasional overlap between accidental, self-inflicted, and potentially externally inflicted mechanisms highlight the diagnostic challenges inherent in these cases. Postmortem imaging is a useful adjunct, but autopsy remains the gold standard for confirming airway obstruction, characterizing associated injuries, and excluding alternative causes of death. Overall, fatal choking in vulnerable populations should be regarded as a preventable cause of death that warrants structured risk assessment, careful medication review, adequate supervision during meals, and close collaboration between clinicians and forensic specialists in a multidisciplinary view.

## Figures and Tables

**Table 1:** Articles included in the revision; first author, reference, title, publication year and article type were schematically reported.

Author	Title	Year	Study Design
Aquila et al. [2]	Could the screening for correct oral health reduce the impact of death due to bolus asphyxia in adult patients? A forensic case report	2018	Case report
Nikolić et al. [3]	Laryngeal Choking on Food and Acute Ethanol Intoxication in Adults-An Autopsy Study	2011	Retrospective study
Bogdanovic et al. [8]	A fatal outcome of PICA syndrome: an usual case of delayed mortality	2018	Case report
D’Ovidio et al. [9]	A particular case of accidental asphyxiation	2018	Case report
De Donno et al. [10]	A singular case of asphyxia by choking on a handkerchief: Accidental event or suicide to “shut-up” spirits	2017	Case report
Busardò et al. [11]	A Very Unusual Accidental Mechanical Asphyxia of Choking with a Whole Solea Solea	2017	Case report
Blaas a et al. [12]	An autopsy approach to bolus deaths	2016	Retrospective study
Salem et al. [13]	An unusual case of smothering by a medical nebulizer	2013	Case report
Redpath et al. [14]	An unusual case of smothering secondary to ingesting raw pet cat	2011	Case report
Saint-Martin et al. [15]	An unusual case of suicidal asphyxia by smothering	2007	Case report
Aquila et al. [16]	Virtopsy versus autopsy in unusual case of asphyxia: Case report	2013	Case report
Pathak [17]	Autopsy detection of café-coronary syndrome in a psychiatric patient	2016	Case report
Wick et al. [18]	Café coronary syndrome-fatal choking on food: An autopsy approach	2006	Retrospective study
Ruschena et al. [19]	Choking deaths: The role of antipsychotic medication	2003	retrospective study
Feola et al. [20]	Choking in patients with neurological disorders and role of drug-induced dysphagia	2024	Case series
Yim et al. [21]	Choking in psychiatric patients: Associations and outcomes	2009	Retrospective study
Deidiker et al. [31]	Return of the killer fish: Accidental choking death on a bluegill (Lepomis macrochirus)	2002	Case report
Byard [23]	Coprophagic cafè coronary	2001	Case report
Jacob et al. [24]	Death, after swallowing and aspiration of a high number of foreign bodies, in a schizophrenic woman	1990	Case report
Mittelman et al. [25]	Fatal aspiration pneumonia caused by an esophageal foreign body	1985	Case report
England et al. [26]	Forensic Features of Pharmacobezoars	2015	Case series
Zuccarello et al. [27]	Forensic investigation on a combined death by food aspiration and acute escitalopram intoxication occurred to a psychiatric subject in a nursing home	2024	Case report
Klein et al. [28]	Homicide or suicide? Xylophagia: A possible explanation for extraordinary autopsy findings	2014	Case report
Nadjem et al. [29]	Ingestion of pointed objects in a complex suicide	2007	Case report
Iino et al. [30]	Postmortem computed tomography findings of upper airway obstruction by food	2010	Retrospective study

**Table 2:** Summary of the data about each case body analysed in the selected articles. (M: Male; F: Female; NA: Not Applicable; ND: Not Determined).

Ref	Number	Age	Gender	Psychiatric Disease	Other Disease	Drugs	Witnessed Death
[2]	1	ND	F	paranoid schizophrenia	NA	antipsychotics and tricyclic anti-depressant	NO
[3]	98	26–92	67 M 31 F	9 schizophrenia, 4 senile dementia, 2 Alzheimer’s disease, 8 psychosis, 9 anxiety, 2 intellectual disability, 1 psycho-organic syndrome (non-hospitalized)	5 psycho-organic syndrome	33: blood ethanol concentration was greater than 5.0 g/dL	15 died in psychiatrists’ facilities; 5 died in healthcare facilities, during therapy
[8]	1	42	M	schizophrenic psychosis	NO	NO	NO
[9]	1	60	M	schizophrenic psychosis	ND	Phenytoin, valproic acid, and phenobarbital, within the therapeutic ranges.	NO
[10]	1	60	F	residual chronic schizophrenic psychosis		Clotiapine 300 mg (3 tablet × 100 mg) and Lorazepam 10 mg (4 tablets tablet × 2.5 mg)	by the caregiver and husband
[11]	1	19	F	schizophrenia	ND	Clotiapine	NO
[12]	59	26–91	38 M 21 F	ND	54 persons, both cardiac and neurological conditions as well as combinations thereof were found.	Alcohol	22 persons died in the presence of others.
[13]	1	43	M	paranoia	asthma attacks treated by Ventoline sprays	antidepressant and tranquilizer drugs in therapeutic level	medical staff
[14]	1	39	F	bipolar disease	ND	olanzapine and lithium	neighbors
[15]	1	30	M	borderline personality disorder	ND	antidepressant, tranquilizer and conventional antipsychotic drugs	medical staff
[16]	1	70	F	familiar-type paranoid schizophrenia		tricyclic antidepressants and antipsychotic drugs	NO
[17]	1	69	M	schizophrenia	tachyphagia	ND	ND
[18]	44	30–96	21 M 23 F	6 schizophrenia, 2 mental impairment, 1 obsessive–compulsive disorder	8 dementia, 4 Alzheimer disease, 4 atherosclerotic cerebrovascular disease, 1 multiple sclerosis, 1 Parkinson disease	drugs in therapeutic level	ND
[19]	70	<40 >71	44 M 26 F	10 schizophrenia, 8 organic psychiatric disorders, and 1 another psychiatric illness		ND	ND
[20]	1	48	M	mild-severe mental disability	perinatal asphyxia, ischemic stroke	antipsychotics (clozapine 37.5 mg/die), benzodiazepines (delorazepam 2 mg/die), antiepileptics (oxcarbazepine 600 mg/die) and beta-blockers (atenolol 50 mg/die)	medical staff
	1	38	F	impairment of intellectual functions, language, and schizo-affective disorders	history of ischemic stroke	antipsychotics (Periciazine 30 mg/die), neuroleptics (Chlorpromazine 50 mg/die);	witnesses
	1	64	F	oligophrenia	hyperphagia and swallowing disorders	anti-parkinson drugs (Levodopa 600 mg/die)	family members
[21]	5	18–87	ND	schizophrenia, intellectual disability, organic brain syndrome, delusional disorder, bipolar affective disorder, and depression	dementia, epilepsy, stroke	antipsychotics, anticholinergics, anticonvulsants, benzodiazepines, antidepressants. Chlorpromazine-equivalent dosages were estimated to amount to 0 to 1400 mg, with a mean of 334 mg per day	medical staff and caregiver
[22]	1	45	M	ND	Ethanol intoxication	NA	friends
[23]	1	74	M	ND	dementia	ND	NA
[24]	1	46	F	depressive schizoid psychosis		NO	medical staff
[25]	1	34	M	mental delay		NA	medical staff
[26]	2	24 and 32	F	bipolar affective disorder, anxiety		ND	NO
[27]	1	89	M	ND	Alzheimer’s Disease hypercholesterolemia, urinary and fecal incontinence, carotid atherosclerosis, gastroesophageal reflux, hiatal her nia, bedrest and cerebral ischemia	escitalopram 10 mg	medical staff
[28]	1		M	Schizophrenia		ND	NO
[29]	1	51	F	depression, suspected histrionic personality disorder	ND	ND	NO
[30]	14	46–84	7 M 7 F	psychiatric problems such as schizophrenia	neurological conditions such as dementia, or encephalopathy.	Citalopram Olanzapine Diazepam Venlafaxine Imipramine Desipramine Valproic acid Chlorpromazine	13 NO—1 another resident’s family

**Table 3:** Summary of the data about each case of choking events analyzed in the selected articles. (NA: Not Applicable; ND: Not Determined).

Ref	Survey	Recovery Site	Circumstantial Data	TC	Autopsy	Histology	Toxicology	Foreign Body
[2]	YES	ND	ND	YES	Bilateral subconjunctival haemorrhages;serious periodontal disease with partial endentulism; material in the oral cavity was also found in the carina and bronchi	Small subpleural and subcardiac petechiae and pulmonar parenchymaenlarged and emphysematous.	Antipsychotics and tricyclic anti-depressants in the terapeutic range	Bread
[3]	ND	20 in healthcare facilities; 49 in private homes; 24 in public places; 7 in restaurants and bars	ND	NO	Bolus in the larynx, completely occluding the laryngeal lumen	ND	33: blood ethanol concentration was greater than 5.0 g/dL	Food bolus
[8]	YES	bedroom floor	treated in a psychiatric hospital for 15 years and his last treatment dated 3 years before his death; previous episodes of eating plastic	NO	conjunctival hemorrhages; several longitudinal scratches on the upper lip. In the lower pharynx, partially obstructing the larynx and thus keeping the epiglottis in an open position. The larynx was left with an impression of the bottle lid. In the stomach a folded PVC bag.	Hypopharynx showed severe nonspecific inflammation and necrosis of epithelium; acute emphysema.	Negative for alcohol and drugs.	Bottle lid with a notched edge
[9]	the house was seen to be in a filthy and messy state, with various objects scattered around the house, and dog excrement. In this general disorder, boxes of anti epileptic drugs	house, lying on a mattress on the floor	NO	NO	Bilateral conjunctival bleeding, ecchymosis on the inside of the upper lip, residues of an indefinite material in the oropharynx, within the larynx and the tracheal lumen	Breakage of the inter-alveolar segments, and alveolar lung edema was noted. Amorphous granular material in some of the alveoli, and endo-alveolar hemorrhaging	Phenytoin, valproic acid, and phenobarbital, within the therapeutic ranges.	Indefinite material: compatible with the absorbent part of a diaper
[10]	YES	In the kitchen lying on the floor close to the fridge	previous suicide attempts driven by evil spirits coming from inside her body. A few months before, she had been found unconscious at home due to attempted suicide, and was therefore hospitalized.	NO	Petechiae in the visceral pleura and epicardium, and visceral congestion.hemorrhagic; infiltration in the soft palate and the soft tissues at the upper portion of the larynx	Multiorgan congestion and subpleural emphysema.	Clozapine overdose (0.9 mg/mL), more than three times the prescribed dosage (300 mg/day).	Paper handkerchief (removed by the emergency medical service staff)
[11]	YES	In the kitchen lying on the floor close to the fridge	NO	NO	Marked cyanosis of the head and the conjunc tivae of both eyes showed focal petechial hemorrhages.marked cerebral edema. The fish adhered to the laryngeal inlet which completely obstructed its access. The fish occupied two-thirds of the length of the esophagus. Its head faced downwards, and its extremity was about 5 cm from the esophageal–gastric junction	Generalized vascular congestion and pulmonary edema with focal area of atelectasia and acute emphysema	Clotiapine, detected the concentrations 0.57, 0.38, and 0.95 mg/L in peripheral blood, urine, and gastric contents	The caudal fin of a fish sole
[12]	NO	NO	NO	NO	The foreign bodies were:3 pharyngeal, 35 laryngeal inlet/laryngeal, 2 tracheal, 3 esophageal, 13 all, 3 not defined	NO	1 BAC < 0.5‰. 21 BAC from 0.73% to 3.90%.	36 Meat, 1 Cheese, 3 Fruit, 3 Vegetables, 1 Cake, 1 Pudding, 14 Others
[13]	YES	in the room of healthcare facilities	was hospitalized without his consent at a psychiatric department because he has attempted suicide by drug ingestion.	NO	Within the cervical oesophagus and completely obstructing the upper airways.	NO	Antidepressant and tranquilizer drugs in therapeutic level	Ventolin Nebulizer
[14]	YES	On the floor of the living room.	She had attempted suicide twice before, once with medication and once with a knife. She was being followed up by a psychiatrist and was prescribed lithium and olanzapine. According to her friends, the victims behavior over the past few days was declining and she had a scheduled appointment with her psychiatrist the morning her corpse was found.	NO	Cat scratches, large piece of adipose tissue and an intact left cat kidney molded to the laryngeal opening, thus obstructing it.lungs congested and edematous	NO	Olanzapine and lithium at therapeutic levels in her blood	Raw cat organs’ fragments
[15]	The room contained a mattress, a pail and a roll of toilet paper. The patient lying. Pellets of toilet paper protruded from his buccal cavity	"esclusion room" of healthcare facilities	Mandatory medical treatment in the psychiatric department of a public hospital. He was known to have attempted suicide by submersion.	NO	Five pellets of white paper measuring 5 cm each, impacting within the laryngo pharynx, the cervical esophagus and the superior part of the trachea. Approximately 250 cm3 of white pellets of toilet paper were found in the stomach	NO	NO	Five pellets of white paper
[16]	YES	In the kitchen lying on the floor	ND	YES	Labial and subungual cyanosis; sub-conjunctival petechiae bilaterally; impairment of the stomatognathic apparatus; at the opening of the trachea, the oral cavity material was also found in the carinal and bronchial seats that became of pultaceous consistency and occluding the respiratory tract.	Histology confirmed the presence of natural material (starch fibres) within the pulmonary alveolus.	Tricyclic antidepressants in therapeutic level	Soft bread
[17]	NO	NO	tachyphagia, he was provided with liquid or semi-liquid meal except that day	NO	Impacted food in the mouth right up to the larynx area. The tracheal lumen was found blocked by food. The respiratory mucosa and lungs were congested and showed petechial hemorrhages over the external surface	Non-significant findings	NO	Solid food
[18]	ND	22 nursing homes, 11 home, 4 restaurants.	ND	NO	The foreign bodies were: 10 supraglottic position, 8 infraglottic and 15 combined infra- and supraglottic	NO	33 cases: blood levels of psychotropic medications (tetrahydrocannabinol, paroxetine, diazepam and chlorpromazine) within therapeutic ranges. 4 cases blood alcohol levels of 0.21% (also with 0.24% and 0.33%, and one had a lower level of 0.01%).	21 mixture of food; 13 single large piece of meat, 10 a banana, scrambled eggs, an apricot, pasta, a peanut butter sandwich, bread, potato chips, a grape and faecal material
[19]	ND	ND	ND	ND	YES	ND	5 cases thioridazine; 3 cases lithium in 3 cases. 3 cases antidepressants (dothiepin, temazepam and diazepam) and anxiolytics 2 cases hypnotics.	31 died following aspiration gastric contents, and 39 from non-aspiration choking deaths (the ‘cafe coronary’)
[20]	NO	rehabilitation center	suffered of constipation and swallowing disorders and his food plane was based on food chopped into small pieces	NO	Filled the larynx occluding the aditus till the first tracheal rings. Also in the first section of the esophagus	Signs of massive broncho-aspiration were represented by small foreign bodies (food materials) in the lumen of the small and very small caliber bronchi and in the alveolar cavities. Food residues were also associated to pulmonary oedema and interstitial emphysema	Delorazepam: 60 ng/mL (10–70 ng/mL); oxcarbazepine: 29 μg/mL (10–35 μg/mL). The toxicological analysis for alcohol was negative	Food bolus cooked and compacted vegetables with meat residues
	NO	nursing home	ND	NO	Inside the oral cavity and the nasopharynx, trachea	Pulmonary oedema and interstitial emphysema	The blood levels of chlorpromazine and piericiazine were 302.3 ng/mL and 27.4 ng/mL respectively. They were both within the therapeutic range (30–300 ng/mL for chlorpromazine and 5–30 ng/mL for periciazine). The toxicological analysis for alcohol was negative.	Grey-brown spherical foreign body with paste consistency;
	NO	sitting in the back seat of the car.	Few years before death a Parkinson’s disease was also diagnosed. She also developed hyperphagia and swallowing disorders. For this reason, her caregiver used to cut solid food in small pieces to avoid chocking.	NO	Laryngeal inlet, esophagus, upper airways. Trachea and bronchi were empty except for small pieces of under-chewed food material on the mucosa.	Pulmonary oedema and interstitial emphysema were present along with signs of anthracosis	Blood levels of levodopa of 1.4 μg/mL within the therapeutic range (0.3–2.0 μg/mL). The toxicological analysis for alcohol was negative.	Small pieces of sandwich
[21]	NO	ND	Hospitalized patients. Choking associated visiting hours	ND	Hypoxic brain damage, stomach distended, trachea and right bronchus contained undigested food	Foof particles im the airspace bronchi and alveol; pulmonary oedema	ND	Varous food (pizza, fruit, cake, etc.)
[22]	ND	A riverbank	After catching a fish, he picked up the fish and He then attempted to swallow the fish whole, headfirst. Moments later, he began gasping and grabbing at his mouth	NO	A fish was identified in the hypopharynx in the proximal esophagus. The body and tail of the fish essentially filled the hypopharynx.	The lungs were markedly congested and showed mild to moderate pulmonary edema	A blood alcohol level of 0.216 g/dl and a vitreous alcohol level of 0.244 g/dl.	A bluegill 13 cm long
[23]	Found dead in bed	found dead in bed	difficulty swallowing and compulsive ingestion of food and other objects, including fecal material	NO	Fecal material was present within his mouth and nose;bolus of impacted feces in laryngopharynx extended to the vestibule of the larynx completely occluding the entrance, upper third of the esophagus	NO	Drugs in therapeutic level	65 g fecal bolus
[24]	NO	NA	At the emergency room for cacute, massively swollen abdomen and severe dyspnea.	YES	The left main bronchus a corroded nail and right main bronchus a screw. The stomach was completely filled with a conglomerate of foreign bodies.	Superficial gastritis with few iron deposits in the mucosa. On the outer stomac wall an extensive purulent fibrinoid inflammation. In the lungs signs of shock.	NO	422 metallic objects: −61 screws; 52 nails; 46 metallic wires and parts of ball point pens; 39 parts of zip fasteners buckles and claps; 37 coins; 34 buttons; 33 keys; 30 paper clips and hairpins; 24 needles; 20 safety pins; 18 rings and parts of jewelery; 9 nuts; 6 curtain peg rolls; 5 parts of wrist watch bands; 4 pictures fasteners; 2 button batteries; 1 wrist watch; 1 bezoar of hair and leather parts
[25]	NO	Room at mental institution	NO	YES	Stuporous and dehydrated, with marked cachexia. Severe tachypnea and dyspnea were noted; extensive multilobular and muhffocal confluent infiltrates with multiple gray foci and consolidations were found in both lungs. in the esophagus: a stone was impacted in the upper esophageal lumen, compressing the trachea from behind	Bronchi, bronchioli and alveolar spaces were filled with foci of suppurative inflammation.	NO	stone
[26]	numerous empty medication packets	Bed at home	Recent depressive episodes reported	NO	Extensive tablet residues were present within the pharynx, esophagus, and stomach with extension into the trachea and bronchial tree, consistent with aspiration. Two pharmacobezoars were present, one within the larynx and another occluding the right main bronchus	NO	High levels of venlafaxine and quetiapine, oxycodone, metoclopramide, topiramate, lorazepam, and loratidine	2 pharmacobezoar
	medication packets	ND	self-harm	NO	The pharmacobezoar tracked into the esopha gus and trachea with extensive involvement of the bronchial tree forming a cast of the airways	NO	Lethal levels of quetiapine and paracetamol and greater than therapeutic levels of lamotrigine and metformin. In addition, therapeutic levels of topiramate, nap- roxen, ibuprofen, and fluoxetine and a subtherapeutic level of diazepam were observed	2 pharmacobezoar
[27]	NO	NO	NO	NO	exogenous material, probably food, up to the bronchial branches	The histological findings of the lungs revealed a dilation of the respiratory spaces, alternating with bronchopneumonia foci, and the presence up to the finest bronchial branches of exogenous material, compatible with liquified food	High concentration of escitalopram in the blood.	food
[28]	There were boards of various sizes, smalsticks, and broken reeds nearby, with no signs of a violent struggle	A riverbank	ND	YES	A large piece of wood, was discovered in the oropharyngeal space. Examination of the mouth, throat, and larynx revealed evidence of the passage of multiple foreign bodies (two branches, various parts of reeds) by mouth into the throat and neck. Foreign body debris in the stomach and intestines	Leukocytosis, microthrombosis, distension, occasional parts of foreign bodies (wood-like) in the bronchi	Antipsychotic and antidepressant in therapeutic level	wood
[29]	YES	bedroom	She was found hanged in her bedroom. Her body was in a sitting position with the back leaning against the bedroom door. A knotted scarf had been used as hanging device forming an open noose which was attached to the door handle.	NO	The local gastric wall showed two perforations by pointed objects. the oesophagus and the stomach showed only minor lesions of the mucosa	Histological investigation of the oesophagus, the stomach and the duodenum revealed multiple minor epithelial lesions of the mucosa surrounded by fresh haemorrhages with emigration of numerous neutrophil granulocytes	Traces of nordiazepam, diazepam and their metabolites (all < 20 ng/mL)	107 pins, 37 nails, 9 sewing needles, 5 safety pins and 12 drawing pins, 7 matches split in half and 2s cent coins.
[30]	NO	In six cases on the kitchen floor. In one case in care facilities	the incidents happened during meal time.	YES	Food items obstructing the airways were detected at autopsy in all cases.	NO	Alcohol and ⁄or drugs were detected in nine of the cases. In five cases, alcohol was detected at high concentrations	The materials found were listed by the pathologists as unspecified (*n* = 5), bread (*n* = 3), meat (*n* = 3), cake (*n* = 1), ‘‘dim sim’’ (meat dumpling, *n* = 1), and grape (*n* = 1).

## Data Availability

No new data were created or analyzed in this study. Data sharing is not applicable to this article.

## Supplementary Materials

The following supporting information can be downloaded at https://www.mdpi.com/article/10.3390/diagnostics16182974/s1, File S1: PRISMA 2020 Checklist.

## References

[1] Manduchi B., Walshe M., Burke É., Carroll R., McCallion P., McCarron M. (2021). Prevalence and risk factors of choking in older adults with intellectual disability: Results from a national cross-sectional study. J. Intellect. Dev. Disabil..

[2] Aquila I., Gratteri S., Sacco M.A., Nuzzolese E., Fineschi V., Frati P., Ricci P. (2018). Could the screening for correct oral health reduce the impact of death due to bolus asphyxia in adult patients? A forensic case report. Med. Hypotheses.

[3] Nikolić S., Zivković V., Dragan B., Juković F. (2011). Laryngeal Choking on Food and Acute Ethanol Intoxication in Adults-An Autopsy Study. J. Forensic Sci..

[4] Samuels R., Chadwick D. (2006). Predictors of asphyxiation risk in adults with intellectual disabilities and dysphagia. J. Intellect. Disabil. Res..

[5] Baldino G., Biondo T., Raffino C., Čaplinskienė M., Vanin S., Spagnolo E.V. (2025). The Chestnut and the Imperfect Crime: A Case Report of Femicide and Staged Road Accident. Diagnostics.

[6] Kaazan P., Seow W., Tan Z., Logan H., Philpott H., Huynh D., Warren N., McIvor C., Holtmann G., Clark S.R. (2023). Deliberate foreign body ingestion in patients with underlying mental illness: A retrospective multicentre study. Australas. Psychiatry.

[7] Page M.J., McKenzie J.E., Bossuyt P.M., Boutron I., Hoffmann T.C., Mulrow C.D., Shamseer L., Tetzlaff J.M., Akl E.A., Brennan S.E. (2021). The PRISMA 2020 Statement: An Updated Guideline for Reporting Systematic Reviews. BMJ.

[8] Bogdanovic M., Alempijevic D., Curcic D., Durmic T. (2018). A fatal outcome of PICA syndrome: An usual case of delayed mortality. Am. J. Forensic Med. Pathol..

[9] D’Ovidio C., Rosato E., Bonelli M., Carnevale A., Marsella L.T. (2018). A particular case of accidental asphyxiation. Med. Sci. Law.

[10] De Donno A., Marrone M., Santoro V., Ostuni A., Cassano A., Grattagliano I., Introna F. (2017). A singular case of asphyxia by choking on a handkerchief: Accidental event or suicide to ‘shut-up’ spirits. Clin. Ter..

[11] Busardò F.P., Mannocchi G., Pugnetti P., Santurro A., Maggiordomo A., Zaami S. (2017). A Very Unusual Accidental Mechanical Asphyxia of Choking with a Whole Solea Solea. J. Forensic Sci..

[12] Blaas V., Manhart J., Port A., Keil W., Büttner A. (2016). An autopsy approach to bolus deaths. J. Forensic Leg. Med..

[13] Haj Salem N., Aissaoui A., Boughattas M., Chadly A. (2013). An unusual case of smothering by a medical nebulizer. J. Forensic Leg. Med..

[14] Redpath M., Sauvageau A. (2011). An unusual case of smothering secondary to ingesting raw pet cat. Am. J. Forensic Med. Pathol..

[15] Saint-Martin P., Bouyssy M., O’Byrne P. (2007). An unusual case of suicidal asphyxia by smothering. J. Forensic Leg. Med..

[16] Aquila I., Falcone C., Di Nunzio C., Tamburrini O., Boca S., Ricci P. (2013). Virtopsy versus autopsy in unusual case of asphyxia: Case report. Forensic Sci. Int..

[17] Pathak A.K. (2016). Autopsy detection of café-coronary syndrome in a psychiatric patient. J. Indian Acad. Forensic Med..

[18] Wick R., Gilbert J.D., Byard R.W. (2006). Café coronary syndrome-fatal choking on food: An autopsy approach. J. Clin. Forensic Med..

[19] Ruschena D., Mullen P.E., Palmer S., Burgess P., Cordner S.M., Drummer O.H., Wallace C., Barry-Walsh J. (2003). Choking deaths: The role of antipsychotic medication. Br. J. Psychiatry.

[20] Feola A., Ciamarra P., Cavezza A., Carfora A., Campobasso C.P. (2024). Choking in patients with neurological disorders and role of drug-induced dysphagia. Leg. Med..

[21] Yim P.H.W., Chong C.S.Y. (2009). Choking in psychiatric patients: Associations and outcomes. Hong Kong J. Psychiatry.

[22] Deidiker R. (2002). Return of the killer fish: Accidental choking death on a bluegill (*Lepomis macrochirus*). Am. J. Forensic Med. Pathol..

[23] Byard R.W. (2001). Coprophagic café coronary. Am. J. Forensic Med. Pathol..

[24] Jacob B., Huckenbeck W., Barz J., Bonte W. (1990). Death, after swallowing and aspiration of a high number of foreign bodies, in a schizophrenic woman. Am. J. Forensic Med. Pathol..

[25] Mittelman M., Perek J., Kolkov Z., Lewinski U., Djaldetti M. (1985). Fatal aspiration pneumonia caused by an esophageal foreign body. Ann. Emerg. Med..

[26] England G., Heath K.J., Gilbert J.D., Byard R.W. (2015). Forensic Features of Pharmacobezoars. J. Forensic Sci..

[27] Zuccarello P., Carnazza G., Salerno M., Esposito M., Cosentino S., Giorlandino A., Sessa F., Pomara C., Barbera N. (2024). Forensic investigation on a combined death by food aspiration and acute escitalopram intoxication occurred to a psychiatric subject in a nursing home. Int. J. Leg. Med..

[28] Klein A., Schröder C., Heinemann A., Püschel K. (2014). Homicide or suicide? Xylophagia: A possible explanation for extraordinary autopsy findings. Forensic Sci. Med. Pathol..

[29] Nadjem H., Weinmann W., Pollak S. (2007). Ingestion of pointed objects in a complex suicide. Forensic Sci. Int..

[30] Iino M., O’Donnell C. (2010). Postmortem computed tomography findings of upper airway obstruction by food. J. Forensic Sci..

[31] Funayama M., Takata T., Koreki A. (2019). A chicking incidents among patients with schizophrenia may be associated with severity illness and higher dose antipsychotics. Gen. Hosp. Psychiatry.

[32] Yayama S., Tanimoto C., Suto S., Matoba K., Kajiwara T., Inoue M., Endo Y., Yamakawa M., Makimoto K. (2017). Analysis of inedible substance ingestion at a Japanese psychiatric hospital. Psychogeriatrics.

[33] Colucci A., Macorano E., De Gabriele G., Marzaioli A., Cristalli A., Introna F. (2024). When something goes wrong. Clin. Ter..

[34] Hägglund P., Gustafsson M., Lövheim H. (2022). Oropharyngeal dysphagia and associated factors among individuals living in nursing homes in northern Sweden in 2007 and 2013. BMC Geriatr..

[35] Papadopoulou P., Porfyri G.N. (2024). Choking as Cause of Death Among the Mentally Ill: A Literature review. Eur. Psychiatry.

[36] Saccomanno S., Saran S., Coceani Paskay L., De Luca M., Tricerri A., Mafucci Orlandini S., Greco F., Messina G. (2023). Risk factors and prevention of choking. Eur. J. Transl. Myol..

[37] Hou W.H., Moo C.C., Kuo T.L., Kuo C.L., Chu S.Y., Wu K.F., Chen L.W., Li C.Y. (2022). Schizophrenia, but not depression or bipolar affective disorder, adds additional risk of aspiration pneumonia among stroke survivors: A national cohort study in Taiwan. J. Psychosom. Res..

[38] Biondo T., Baldino G., Burrascano G., Tarzia P., Indelicato M., Asmundo A., Ventura Spagnolo E. (2024). Sudden Unexpected Death in Epilepsy (SUDEP). Full Forensic Approach in Two Cases and Review of Literature. Clin. Ter..

[39] Robertson J., Chadwick S., Baines D., Emerson E., Hatton C. (2018). People with intellectual disabilities and dysphagia. Disabil. Rehabil..

[40] Kulkarni D.P., Kamath V.D., Stewart J.T. (2017). Swallowing Disorders in Schizophrenia. Dysphagia.

[41] Robertson J., Chadwick D., Baines S., Emerson E., Hatton C. (2017). Prevalence of Dysphagia in People With Intellectual Disability: A Systematic Review. Intellect. Dev. Disabil..

[42] Luykx J.J., Tanskanen A., Lähteenvuo M., Manu P., Correll C.U., Hasan A., Lieslehto J., Taipale H., Tiihonen J. (2024). Dopamine D2 receptor antagonism of antipsychotics and the risk of death due to choking. Psychiatry Res..

[43] Miarons M., Rofes L. (2019). Systematic review of case reports of oropharyngeal dysphagia following the use of antipsychotics. Gastroenterol. Hepatol..

[44] Fioravanti M.P., Miyahara F.B., Cavallari H.H., Bretan O. (2011). Bedside assessment of swallowing in elderly subjects using psychotropic drugs. Braz. J. Otorhinolaryngol..

[45] Cicala G., Barbieri M.A., Spina E., de Leon J. (2019). A comprehensive review of swallowing difficulties and dysphagia associated with antipsychotics in adults. Expert. Rev. Clin. Pharmacol..

[46] Aldridge K.J., Taylor N.F. (2012). Dysphagia is a common and serious problem for adults with mental illness: A systematic review. Dysphagia.

[47] Sugisawa S., Nozue S., Kurihara T., Koya H., Tsuneoka T., Nagai T., Kurata N., Inamoto A., Takahashi K., Sasaki T. (2020). Asphyxia risk factors in adult psychiatric wards. Perspect. Psychiatr. Care..

[48] Herzig S.J., LaSalvia M.T., Naidus E., Rothberg M.B., Zhou W., Gurwitz J.H., Marcantonio E.R. (2017). Antipsychotics and the Risk of Aspiration Pneumonia in Individuals Hospitalized for Nonpsychiatric Conditions: A Cohort Study. J. Am. Geriatr. Soc..

[49] Camatti J., Santunione A.L., Bolognini M., Cusack D., Zerbo S., Argo A., Puntarello M., Scalzo G., Paolo F., Bruscagin T. (2025). Towards a standard of scientific evidence in on-site inspection: Compilation of the ECLM on-site inspection form in a broad case history. Leg. Med..

[50] Stassi C., Mondello C., Baldino G., Cardia L., Gualniera P., Calapai F., Sapienza D., Asmundo A., Ventura Spagnolo E. (2022). State of the Art on the Role of Postmortem Computed Tomography Angiography and Magnetic Resonance Imaging in the Diagnosis of Cardiac Causes of Death: A Narrative Review. Tomography.

[51] Baldino G., Mondello C., Sapienza D., Stassi C., Asmundo A., Gualniera P., Vanin S., Ventura Spagnolo E. (2023). Multidisciplinary Forensic Approach in “Complex” Bodies: Systematic Review and Procedural Proposal. Diagnostics.

